# Intelligent action guidance and the use of mixed representational formats

**DOI:** 10.1007/s11229-018-1892-7

**Published:** 2018-08-16

**Authors:** Joshua Shepherd

**Affiliations:** 1 Carleton University, Ottawa, Canada; 2 Universitat de Barcelona, Barcelona, Spain

**Keywords:** Action guidance, Representational format, Object perception, Interface problem

## Abstract

My topic is the intelligent guidance of action. In this paper I offer an empirically grounded case for four ideas: that [a] cognitive processes of practical reasoning play a key role in the intelligent guidance of action, [b] these processes could not do so without significant enabling work done by both perception and the motor system, [c] the work done by perceptual and motor systems can be characterized as the generation of information (often conceptually structured information) specialized for action guidance, which in turn suggests that [d] the cognitive processes of practical reasoning that play a key role in the guidance of intelligent action are not the abstract, syllogistic ones philosophers often treat as the paradigm of practical reasoning. Rather, these cognitive processes are constrained by, and work well with, the specialized concepts outputted by perception and the feedback outputted by sensorimotor processes.

## Introduction, with a brief word about cognition and perception

My topic is the intelligent guidance of action. My motivating thought is that recent work examining traffic at the borders between perception and cognition, and cognition and action, is useful for our understanding of action’s intelligent guidance, and of the respective roles of perception, cognition, and the motor system therein. In particular, it seems to me that this work makes available a more nuanced picture of the respective roles played by perceptual, cognitive, and motor processes, and how they interface.

Central to what I have just called a ‘more nuanced’ picture are the ideas that [a] cognitive processes of practical reasoning play a key role in the intelligent guidance of action, [b] these processes could not do so without significant enabling work done by both perception and the motor system, [c] the work done by perceptual and motor systems can be characterized as the generation of information (often conceptually structured information) specialized for action guidance, which in turn suggests that [d] the cognitive processes of practical reasoning that play a key role in the guidance of intelligent action are not the abstract, syllogistic ones philosophers often treat as the paradigm of practical reasoning. Rather, these cognitive processes are constrained by, and work well with, the specialized concepts outputted by perception and the feedback outputted by sensorimotor processes.

Making the case for these ideas and the relations between them is the task I set myself in this paper. Before I begin, however, I wish to address a set of issues that lingers in the background. Above and below I talk freely of ‘cognitive’ processes and ‘perceptual’ processes. One might worry that such usage is problematized by recent disputes about the nature of cognition, the viability of proposals regarding a ‘mark of the cognitive,’ and whether cognitive science in fact needs a mark of the cognitive (e.g., Adams and Aizawa 2001; Buckner 2015; Allen 2017), as well as disputes about how we might draw a distinction between cognition and perception, and whether any such distinction is viable (see Shea 2015; Burnston 2017a; Beck 2018; Phillips 2017). Such disputes might reasonably raise questions about my usage of ‘cognitive’ and ‘perceptual.’ Some clarification regarding my usage might be helpful.

I take it standard usage characterizes paradigmatically perceptual processes as those essentially bound up in the production of stable, objective, largely veridical representations of the world in particular sensory modalities such as vision or audition, or in combinations of modalities. Such usage permits significant unpacking and refinement, of course. In this connection, Ben Phillips (2017) persuasively argues that though the border between perception and cognition can be characterized in a plurality of ways, the most perspicacious of these ways will involve the notion of stimulus control (see also Beck 2018). So, perceptual processes can be grouped as processes the function of which is to represent environmental entities in a way that is under the causal control of ‘some unique stimulus-type or combination of stimulus-types’ (Phillips 2017, p. 11). This characterization builds fruitfully upon standard usage regarding paradigmatic perceptual processes, and, as Phillips argues, offers illumination regarding problem cases (e.g., visual short term memory, perceptual demonstratives).

Cognitive processes will not share this function. Rather, borrowing here Buckner’s (2015) helpful list, paradigmatically cognitive processes include or involve ‘conceptual abilities, cognitive mapping, transitive inference, episodic memory, numerical competence, sequence and serial position learning, causal inference, analogical reasoning, language use, imitation, mindreading, and metacognition’ (Buckner 2015, p. 309). While it is an open question whether such processes form anything like a natural kind, Buckner renders plausible the idea that cognitive processes form a systematically correlated cluster that share a number of important properties. Central to the shared properties is the function of enabling adaptive, efficient, and context-sensitive shaping of behavior (Buckner 2015).

I am obviously not offering a resolution of debates about the nature of perception, or of cognition, or of the border between them. My aim is clarity regarding the usage of the terms in this paper. When I talk of cognition using perception, I am talking of processes and capacities not under direct environmental stimulus control, the function of which is context-sensitive behavior shaping (‘the cognitive’), and I am talking of processes and capacities the function of which is to represent environmental entities in a way that is under stimulus control (‘the perceptual’). Of course it remains possible that there is neither a mark of the cognitive (but see Buckner 2015), nor a viable distinction between cognition and perception (but see Beck 2018; Phillips 2017). If that turns out to be the case, what I say below should be re-interpreted in terms of the more specific capacities, processes, and tasks at issue, rather than in terms of the more general distinction between perception and cognition.^1^

## Representational formats

Psychological states not only have representational content, but encode this content in a representational format. A format can be thought of as a representational system specified in terms of a proprietary set of representational primitives and combinatorial principles. Different representational systems function as kinds of languages, capable of expressing kinds of contents. So, for example, representations to do with audition, with vision, with multi-modal perception, with sentences of a human language (perhaps in inner thought), with maps, with what is sometimes called an individual’s action semantics (or motor grammar), with analogue magnitude representations, with phonological structure in linguistic processing, with syntactic structure in linguistic processing, and with maps used in navigation are all at least candidate examples of differently formatted psychological states.

Differences in representational format mark important differences in psychological functionality. For present purposes, at least four differences deserve mention. First, differences in representational format mark restrictions on the content individual psychological states can carry. Call this a *state*-*content restriction*. So, for example, an analogue magnitude representation can represent a magnitude of some amount, but not an integer (Beck 2015).

Second, differences in representational format mark restrictions on the content a representational system can carry. Call this a *system*-*content restriction*. An individual’s motor grammar helps compose a kind of semantics for action execution, but motor representations cannot represent items that sentences can—e.g., facts about quantum mechanics, or Cousin Stizz’s latest mix tape, or Jack Lyons’s excellent work on iconic representations.

Third, due to these restrictions on content, and in some cases due to differences in combinatorial principles, differences in representational format mark differences in the content-driven transitions available within a format. So, for example, both Camp (2007) and Rescorla (2009) have argued that sets of map-like representations permit a range of systematic transitions, even though these transitions fall short of those allowed by fully discrete (e.g., linguistically structured) representational formats. Beck (2015) has argued for something similar regarding analogue magnitude representations. And arguably, pictorial representations cannot be used to assert or negate anything (see Crane 2009 for this view, and Grzankowski 2015 for an argument against it).

A fourth difference concerns, not the transitions a format enables and rules out in some absolute sense, but in a more specific sense the *manner* of transitions a format enables and rules out. Some transitions will be made quicker and others slower, for example—a critical feature of a format’s *usability* by time-sensitive creatures like humans. Consider, in this connection, Camp’s discussion of a difference between map-like and sentence-like representations:

A map can easily represent the locations of and relations among many objects and properties in an explicit yet cognitively transparent way, thereby minimizing the need for processing to recover those locations and relations. By contrast, it would be massively cumbersome to spell out this same information in sentences: a practically feasible sentential representation will only specify some of that information explicitly, and will rely on processing to make latent information explicit. However, because the number of further sentences one can derive from any substantive set of initial premises is so large, it’s not feasible to just crank out that information by brute force. For practical purposes, a thinker needs a content and context-sensitive way to extract relevant information. Thus, when dealing with relative spatial locations, sentential systems face a processing challenge, and a risk of processing error, that cartographic systems don’t. (2007, p. 162)

Given these differences, a proper characterization of a state’s, or a system’s, representational format(s) seems a critical task for psychological theory. As mentioned in section one, in this paper I am primarily concerned to explore ways that different representational formats may enable explanations of intelligent action guidance. With this in mind, I turn to recent work on the formats involved in certain kinds of perception.

## Object perception

Earlier I mentioned the literature addressing the putative border between perception and cognition. One common way to distinguish between perception and cognition—though not a way I take here—appeals to representational format. The idea is that perception deals in iconic (or analog) representations, and cognition deals in discursive (or digital) representations. Iconic representations follow two principles that Green and Quilty-Dunn (2017) call ICONICITY and HOLISM:

ICONICITY: Parts of the representation represent parts of the scene represented by the whole representation.

HOLISM: Each part of the representation represents multiple properties at once, so that the representation does not have separate vehicles corresponding to separate properties and individuals. (2017, p. 11)

A representation that follows these principles cannot help but pack in lots of information—whether it’s germane at a time or not. Consider, for example, Dretske’s example of representations involving coffee.

If I simply tell you, ‘The cup has coffee in it,’ this (acoustic) signal carries the information that the cup has coffee in it in digital form. No more specific information is supplied about the cup (or the coffee) than that there is some coffee in the cup. You are not told how much coffee there is in the cup, how large the cup is, how dark the coffee is… If, on the other hand, I photograph the scene and show you the picture, the information that the cup has coffee in it is conveyed in analog form. The picture tells you that there is some coffee in the cup by telling you, roughly, how much coffee is in the cup, the shape, the size, and the color of the cup, and so on. (1981, p. 137)

As Quilty-Dunn (2016) has noted, if representations utilized by perceptual processes violate these principles, we have reason to think that perceptual processes do not traffic exclusively in iconically formatted representations.

In this connection, Quilty-Dunn highlights research utilizing the following experimental paradigm. Experimenters place participants in front of a computer screen, on which objects are identified with markers, such as letters. The markers disappear, the objects move around in space, the markers are re-presented (either attached to the previously associated objects or not), and the participants are required to identify the re-presented markers. It turns out that subjects are quicker to identify the markers when they are re-presented on the objects with which they were originally associated. This is called the object-specific preview benefit.

This object-specific preview benefit has been fruitfully explained by the posit of object files (Kahneman et al. 1992)—that is, a representation in visual short-term memory capable of encoding and attributing a range of features to an object. Quilty-Dunn (2016) argues that object files do not behave in ways iconically formatted representations must. In particular, object files seem capable of encoding and attributing a too-flexible range of features to objects. These include low-level properties such as shape and color, as well as ‘which letter appears with the object even independently of typeface and case (Henderson 1994), that the object is labelled as a fish even independently of whether the label is the word ‘fish’ or a picture of fish (Gordon and Irwin 2000), and that the object is labelled as a piano even independently of whether the label is a picture of a piano or the sound of a piano being struck (Jordan et al. 2010)’ (260). The representational flexibility of object files is inconsistent with a view on which perception outputs only iconically structured representations. Object perception processes appear to construct, utilize, and output discursively structured representations of objects.

A second relevant experimental paradigm requires subjects to categorize items under extreme time constraints. In one study (Potter et al. 2014), subjects are asked to look for something—say, a flower—and are then presented with a series of images, and then asked whether they had seen a relevant (e.g., flower) image. (In some conditions, subjects were given 12 images in a row—too many for them to hold in working memory.) If they answered yes to the relevant prompt, the subjects were shown two flowers and asked which one it was. Remarkably, subject performance was above chance even when items were presented for 13 ms. As Mandelbaum (2017) has argued, this result (and many others within this research paradigm) would be extremely puzzling if ‘perception has to first output a wholly non-conceptualized representation and then match that representation to a concept in central cognition’ (5). For 13 ms is far too quick for information to travel from perception to any other kind of categorization mechanism and back.

One might worry, however, that the 13 ms presentation time is misleading. Might whatever representation is developed on the back of the 13 ms of processing be stored or transmitted to cognition, for use or further processing by cognition, leading to the answers participants are able to give? If so, there would be reason to doubt Mandelbaum’s interpretation. Consider, however, a study by Grill-Spector and Kanwisher (2005), that Mandelbaum highlights. In this study subjects were split into three conditions. In the first, subjects were asked to discriminate between a rapidly presented target image or rapidly presented visual noise (rapid = 17 ms). The experimenters called this a detection task. In a second, subjects were asked to identify certain images out of a series of rapidly presented images (e.g., a car). The experimenters called this a categorization task. In a third, subjects were asked to identify something more specific—whether a subordinate category (e.g., a Jeep as opposed to simply a car) had appeared. The experimenters called this a within-category identification task. Even at 17 ms, subjects did well, and performed similarly, at the first two tasks. They performed poorly, and took longer, on the third. Regarding the present worry, the similar performance in the categorization and detection tasks is relevant. Grill-Spector and Kanwisher offered two possible explanations for this similarity. First, ‘detection and categorization require the same amount of time’ (2005, p. 154). Second, ‘the same amount of stimulus information is necessary for detection and categorization, but categorization requires additional processing’ (154). To help determine which one might be correct, Grill-Spector and Kanwisher performed an additional study that measured performance as well as reaction time. They report that ‘that not only accuracy but also RTs were virtually identical for the detection and categorization tasks… In contrast, RTs were longer for the identification task, even when accuracy in categorization and identification were matched’ (155). The inference to draw is that object detection and object categorization require the same amount of processing time—an inference that runs against the worry that later, arguably cognitive, processing is partially responsible for participant success at categorization.

Return, now, to the difference between accuracy and response time between detection and categorization on the one hand, and subordinate-level identification on the other. Regarding this difference, Mandelbaum asks a good question, and provides a compelling answer.

What would explain why detecting or categorizing a basic-level stimulus is equally easy, while merely detecting a subordinate exemplar is considerably more demanding? This pattern of behavior should be expected if the output of the modular process is in fact a basic-level concept, like DOG, FLOWER, and HOUSE. If perception outputs basic-level concepts then detecting the presence of the basic-level content would be no harder than categorizing because perception itself would accomplish both tasks. Since one has no top down access to the workings of the modules, even though detection would (presumably) occur first, response times and accuracy rates would not be affected because the rest of cognition only has access to the outputs of perception and those outputs would arrive already categorized for basic-level concepts. Conversely, response times spike and performances decline on the subordinate task because that task involves identifying subordinate-level categories and these subordinate-level categories are *not* the proper outputs of perception. That is, they are outside the bounds of perceptual content. (2017, p. 8)

Say that this picture of object perception is accurate. Perception outputs discursively structured object files, at least regarding basic-level concepts. Why would it do so? Mandelbaum suggests that ‘basic-level categories would be outputted by perception for that is the level of categorization that allows for easy action’ (10). This suggestion evokes the thought that the conceptualized outputs of perception feed directly into motoric guidance systems—perhaps via the dorsal stream (Milner and Goodale 2008). Even if this is right, an interesting question arises regarding outputs to cognitive processes. Might the perception of objects—and if events are simply the possession of various properties by objects at or over times, we could consider this data as relevant to event perception as well—play some special role in the way that cognition *uses* perceptual representations?

In connection with this thought, notice that the claim that perception outputs conceptualized or discursive representations leaves much room for further characterization of the representational format of these psychological states. All that is being claimed here is that these representations possess some non-iconic structure that enables the demonstrated behavior, i.e., an object-specific preview benefit, or the rapid and accurate categorization of sensory stimuli. Perhaps object and event files should be thought of as propositionally structured (Green and Quilty-Dunn 2017). Even so, it is dubious that the structure of these representations is *atomic* like that of Fodor’s (1998) lexical concepts, i.e., that these representations possess no internal structure that could help determine their referents. Plausibly, such representations attribute properties to objects, where these properties are at least partially coded in a sensory or pictorial format. After all, in the Potter et al. study discussed above, participants could identify the object not only as a flower, but as the particular flower that it was. Although the possibility requires further analysis and experimentation, it might be that (like analog magnitude representations (see Beck 2015)) these representations possess a discursive but proprietary structure permitting some transformations and constraining others.

I return to these issues below. Our present question is how the outputs of perception might play a role in (constraining or enabling or influencing) the way that cognition *uses* perceptual representations, in particular in guiding intelligent action.

## Cognitive use of perceptual representations

One might think that in concluding the previous section with a question about cognition using perceptual outputs for action guidance, I am moving too fast. Why think cognitive processes guide action? In particular, given the very rapid categorization results referenced above, why not think the outputs of perception run directly to motoric processing—that perception is what directly guides action? After all, decades of science indicate that the existence of a processing stream within vision—the dorsal stream—directly connects to motoric processing, and is functionally specialized for guiding action (Milner and Goodale 2008).

I would not deny the existence of a functionally specialized visuo-motor—or even perceptuo-motor—stream of information. But functional specialization is not the same thing as informational encapsulation (Ferretti 2017). Rich connections exist between the dorsal stream and the ventral stream—the stream regarded as functionally specialized for object recognition, and more closely connected to conscious visual experience. Furthermore, although the dorsal stream is functionally specialized for enabling fine grained visuo-motor transformations and for enabling very rapid on-line motor corrections, the data suggests that conscious vision and processing in the ventral stream is required for normal action guidance (see Shepherd 2015; Zipoli Caiani and Ferretti 2017), as well as for actions in a wide range of contexts—e.g., slightly slower visually guided actions, unfamiliar actions, and so on. I cannot review all of the evidence here, but Briscoe and Schwenkler (2015) have done so, convincingly concluding that ‘there is no general, empirically based objection to the notion that consciously encoded information is used to control bodily actions’ (1460).

This clears the air somewhat. But Briscoe and Schwenkler are primarily focused on the role of conscious visual representations in action guidance. But we want to know about the role of cognitive processes in using such representations. In this connection, consider the following suggestive study by Creem and Proffitt (2001). They demonstrated that the execution of an appropriate grip action can be undermined by giving a participant a competing cognitive task to perform. They had participants perform an action of picking up and placing an object onto a table. The appropriate grip for this action was to reach around the object in order to get at the handle. The participants performed this task alone, or alongside the performance of a spatial imagery task, or alongside the performance of a challenging ‘semantic’ task that involved hearing, remembering, and re-stating certain words. It turns out that participants were much worse at executing the appropriate grip when cognition was taxed via the semantic task, but not via the spatial imagery task. Creem and Proffitt reason as follows:

When participants were asked to pick up objects without a secondary task, they most often reached around to pick the objects up by their handles in a manner appropriate for use of the objects. However, when the cognitive system was taxed by a concurrent semantic task, they rarely picked up the objects appropriately. The semantic task may have interfered with the depth to which participants could semantically process the objects and, thereby, limited the information made available to the visuomotor system about where to grasp the object. Furthermore, the results from the spatial dual-task condition suggest that the interference was specific to the semantic system responsible for identifying the object and its functional qualities. There was little decrement in performance when the spatial task was performed concurrently. (221)

What is the relevance of this result? Consider what you might say if you thought that the kinds of states cognition worked with were completely distinct in representational format from the outputs of perception, or if you thought that perceptual and motor processes were sufficient for guidance. This result would be odd—because here we see cognition is involved even in mundane object analysis, even in mundane action guidance, and in the working through of how best to accomplish the goal.

This gives some indication of the involvement of cognition in action guidance. But the information given is admittedly sparse. We need a more perspicuous characterization of how cognition uses perceptual representations.

Consider a study by Bekkering and Neggers (2002) in which participants were instructed to search for and find predefined targets. In one condition, participants were told to point to the target. In another, participants were told to grasp the target. In the latter condition, of course, participants would have possessed a target-relevant conditional intention. In that condition, participants made fewer erroneous saccades to non-target objects. As Bekkering and Neggers explain, ‘The results suggest that a specific action intention, such as grasping, can enhance visual processing of action-relevant features, such as orientation’ (370). Note that in this case what is aided is a perceptual process (visual search and detection) explicitly guided by cognition (including the cognitive state of intention).

An interesting series of studies by Humphreys and Riddoch (2001) corroborates this thought. Their studies involved a patient (MP) with an injury leading to unilateral neglect: the patient was unable to find targets when the targets were described by a name or by visual properties (e.g., color). Even so, this patient was able to find targets when they were described by performable actions—as, e.g., something one could drink from, or write with. Importantly, this result does not appear to be entirely due to object perception processes: MP was just as good at detecting actionable novel objects invented by experimenters as MP was at detecting familiar actionable objects (study 6). It appears, rather, that MP retained the ability to use a proprietarily actional template to guide visual search. Humphreys and Riddoch explain:

Our results imply that search can be based on intended actions, and not just on the perceptual properties of objects. MP could name individual objects and colors, but he had difficulty with these cues in search. This finding suggests an impairment in linking perceptual cues in memory to objects, when multiple objects compete for attention. In contrast, MP could detect the same targets when cued by action. This indicates that action templates can influence visual search and selection independently of perceptual templates of targets. (86)

The picture that is emerging makes room for cognitive, action-relevant states and processes (intentions, action-planning)—states and processes that operate over discursively structured representations—interacting in close ways with perceptual states and processes (spatial attention, object detection) to support action.^2^ Importantly, while these states and processes seem to share structure with (and thereby to guide) perceptual states and processes, they are also possessed of the modal profile of paradigmatically cognitive states and processes—that is, these states and processes are explicitly and flexibly utilized and deployed by agents, suggesting that agents possess a degree of flexibility and cognitive control with respect to them.

These points might be thought to apply only locally, i.e. to processes of perceptual search or perhaps attentional guidance. I think, however, that they fit nicely within a broader account of intelligent action guidance. To say why, I turn to the role of processes of practical reasoning in action control.

## Action concepts and practical reasoning

We are stalking an account of how cognition uses perceptual and motor representations. I offer two ideas. First, the use can be reconstructed as a process of practical reasoning.^3^ Second, the process of practical reasoning is both enabled and constrained by the formats of the states involved. In many cases, these will be mixed formats.

Take the first idea first. I understand practical reasoning, roughly, as psychological processing that can be usefully interpreted as directed towards behavior production, and that consists in transitions between representational states and attitudes, where these transitions are sensitive to norms of practical rationality (or, if one prefers, to practical reasons). Why think the cognitive use of perceptual and motor representations to guide action can be construed in this way?

Consider the following picture of action guidance, offered in a recent paper by Mylopoulos and Pacherie (2017). Actions are generated and sustained by intentions, which are often (though not necessarily) formed by processes of practical reasoning. The intentions that guide on-line action execution are proximal intentions, and these have an integrative function with respect to incoming perceptual information and stored knowledge about the action: intentions ‘integrate conceptual information about the intended action… with relevant perceptual information about the current environment in order to yield a more specific situated representation of the action to be performed’ (321). What, then, is the representational format of an intention? Mylopoulos and Pacherie make two relevant comments. First, that ‘the content of proximal intentions cannot be purely descriptive, it must be at least in part indexical and include pointers to elements of the environment’ (321). Second, they stress a propositional representational format, and the role this plays in enabling rational action guidance:

[C]oncepts are the inferentially relevant constituents of intentional states and… their sharing a common conceptual representational format is what makes possible a form of global consistency, at the personal level, of our desires, beliefs, intentions and other propositional attitudes… what follows is that for intentions to satisfy the rationality constraints they are subject to, they must have a propositional format and conceptual content. (321)

Intentions contain conceptual, propositionally formatted content about actions. But not just any kind of conceptual content will do. Mylopoulos and Pacherie identify a special kind of action concept—an executable action concept—that ‘hooks up’ in some way with the motor representations that implement fine-grained details of action execution.

On this picture, then, intentions—or the processes that construct them—integrate the conceptual, rationally structured information that an agent brings to an action with the perceptual information required to set parameters for action execution. And this conceptual information is not purely abstract (or amodal), although it is propositionally formatted. For this information is linked with an agent’s abilities—‘If one has an executable action concept, then one has the ability to perform the action in question’ (329).

But why link the concept and the ability in this way? Mylopoulos and Pacherie do so because of a felt need to address the ‘interface problem’ (Butterfill and Sinigaglia 2014)—the issue of explaining how propositionally formatted intentions coordinate with motorically formatted motor representations. On their view, the possession of executable action concepts requires the possession of more fine-grained representational states that could guide action, such as motor schemas. It is at this point, however, that Mylopoulos and Pacherie have been criticized (by, e.g., Burnston 2017b; Shepherd 2017). The worry is that we still lack an explanation of how these differently formatted representational states coordinate to enable (or partially constitute) the ability to intelligently guide action.

In previous work I have argued that we can make progress on the interface problem by conceiving of the relationship between intentions and motor representations in a certain way. My argument relies in part on the following experimental result (plus others, of course: see Shepherd 2017). Day et al. (2016) utilized a paradigm in which participants are asked to perform aiming actions towards seen targets located around a circle, under a condition of rotated (non-veridical) feedback. In this case, participants were asked to report their aiming location (their intention) before each action. Occasionally, after performing a series of such actions, participants would be asked to aim at a target with all aiming landmarks and visual feedback removed. Day et al. added these trials into the experiment in order to measure the progress of implicit motor learning—measured as the difference in location between the actual movement and the intended action.

Consistent with other work, Day et al. found implicit learning in the task. Once given time to process the non-veridical feedback on several trials, participants moved farther away from their intended location in a direction that suggests sensorimotor learning processes were implicitly trying to correct for the apparent errors made. This result, along with many others, confirms a picture on which processes of practical reasoning and intention formation are somewhat distinct from processes of sensorimotor execution and adaptation. For the sensorimotor processes operate according to their own principles.

Even so, the Day et al. results suggest there remains a close connection between intentions and the sensorimotor processes. For Day et al. found that differences in the amount of implicit learning were based upon the intentions participants reported, rather than their actual movements. Day et al. explain:

[A]s participants aimed farther from their most frequently reported aiming location, the magnitude of implicit learning decreased. Thus, implicit learning generalized maximally at each individual’s most frequent aiming location and decays as a function of angle away from that aiming location. (7)

Implicit learning generalized around the intended location and not the actual location. Day et al. reason as follows:

There is obvious interplay between the cognitive and implicit processes involved in motor adaptation… the two are not merely engaged in a simple give-and-take relationship to achieve task goals, but rather the implicit sensorimotor recalibration that defines visuomotor adaptation is learned around the cognitive representation of the movement. (11)

Why think this result regarding sensorimotor adaptation generalizes to action control in broader contexts? First, studies subsequent to the Day et al. study replicate the result while extending it in the direction of a general model of sensorimotor adaptation that incorporates a role for explicit planning and intention formation processes (McDougle et al. 2017). What’s more, these processes of planning appear to be important for a critical aspect of skill learning, namely, the individuation of motor memories. Sheahan et al. (2016) utilized an experimental design that required participants to deploy distinct, explicitly formulated plans for movement. The movements involved were very similar and would normally show high interference effects if directed towards different tasks. Interestingly, attaching these similar movements to distinct explicitly formulated plans led to a reduction of interference. As they put it, ‘the key to representing multiple [motor] memories is to have each associated with a different motor plan’ (2016, p. 775).

These recent studies should raise our confidence in the importance of explicit planning, reasoning, and intention formation processes in the structuring of motor learning, and thereby motor execution. Additional support for this idea comes from the fact that these studies are consistent with—and can be seen as a more specific elaboration of—a well-confirmed model of the psychological architecture underpinning action control (for discussions that emphasize and develop this picture in various ways, see Fridland 2014; Shepherd 2015; Christensen et al. 2016). That architecture is hierarchical, moving from more abstract representations and processes to more specific. That architecture also allows for significant interaction between levels, and significant guidance of processing at more specific levels by processing at more abstract levels. As Christensen et al. put this point, ‘controlled and automatic processes are closely integrated in skilled action, and that cognitive control directly influences motor execution in many cases’ (2016, p. 43). There remain questions about how exactly cognitive control processes map onto lower levels in the action control hierarchy in particular cases. One thing recent work on sensorimotor adaptation suggests is that the mapping is subserved by mechanisms within motor control that are sensitive to, and operate over, the content of higher level processes.

There are reasons to think, then, that the Day et al. study points to, and coincides with, a fundamental truth about the psychological architecture of action control. Sensorimotor adaptation processes take on board elements of an intention’s content in performing their characteristic functions. How do they do so? I have argued for the following explanation. Agents are capable of linking the conceptual components of states like intentions with the motoric components of motor representations within cognition: agents have the ‘ability to put propositional level action understanding and (some aspects of) motoric level action implementation together within explicit practical reasoning’ (Shepherd 2017, p. 16).

This suggestion requires further explication. One way to further develop this basic idea is to maintain that the representational processes at issue in the above experimental paradigm, and at issue in intelligent action guidance more generally, involve psychological states with mixed representational formats. I explicate and defend this idea in the next section.

## Mixed formats

Although the notion of mixed representational formats has received little explicit discussion in the literature,^4^ it should be somewhat familiar. Many maps we readily use are a mix of a map-like format and a linguistic format. Consider, for example, two maps, A and B. As a map normally does, A operates partially via an isomorphism between its spatial layout and the spatial layout of the region A represents. Say that A represents the layout of some space. Now consider B, which represents the layout of the same space, but contains content that goes beyond what maps proprietarily represent: say content regarding specific people. My idea here comes from a map suggested by Camp (2007):
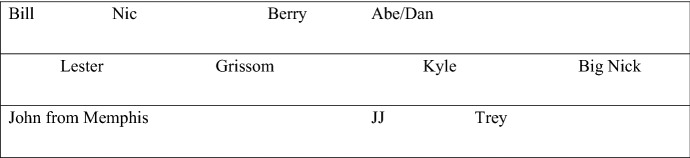


This could be a map of a room at a party, or of the configuration of players in some game. Whatever the case, once you embed conceptual information about the particular items on the map—the people—a wider range of transformations are permitted. You might decide to avoid the part of the room that contains John from Memphis and Lester, because of an inference you draw about those two in close proximity to each other. In this way the mixed format map is more useful than a map that failed to include information beyond location, distance, relation to nearby locations, and so on.

It is, of course, one thing to consider mixed format representations explicitly created for human use. It is another to consider whether any psychological mechanisms function by using mixed format psychological states. What I want to suggest is that cognitive processes of practical reasoning do make use of such states.

Suppose, then, we committed to the idea that processes of practical reasoning utilized mixed format states. To what would we be committing? One’s answer might depend on one’s account of how to individuate representational formats. Above I suggested that a representational format can be characterized as a proprietary set of representational primitives and a set of combinatorial principles. This characterization makes it difficult to distinguish between a process that utilizes mixed format states and a process that integrates or maps content from one or more formats into another.

We do, however, have suggestive evidence that cognition does not always map content delivered from the senses into any proprietary kind of code. It is a central lesson of the concepts literature that paradigmatically cognitive, conceptual tasks are often performed in ways that suggest the processes at issue traffic in modality specific—sensory or sensorimotor—representational formats (Barsalou 2008; Dove 2009).

Consider one result among many. Glenberg and Kaschak (2002) had participants judge whether written sentences were sensible (e.g., ‘‘Open the drawer’’) or nonsense (e.g., ‘‘Open the plate’’). One set of sentences suggested action moving toward the participant (e.g., ‘‘Open the drawer’’). Another set of sentences suggested action moving away from the participant (e.g., ‘‘Close the drawer’’). Participants would give their responses in one of two manners: by moving their hand away from the body to push a button, or by moving their hand toward the body to push a button.

Given that set-up, what might we predict? Glenberg and Kaschak (2003) consider two relevant hypotheses. According to the first, ‘Language is understood using a system of amodal symbols, but then is translated into a response code for guiding action’ (97). According to the second, ‘Language is understood directly in terms of action’ (97). They argue that if the second hypothesis is right, ‘the same neural systems used to plan and guide action are also used to comprehend language’ (97). On that hypothesis, one might predict that ‘the mere understanding of a sentence should facilitate (or interfere with) congruent (or incongruent) action, and similarly, physical action could facilitate or interfere with understanding’ (97). This is what Glenberg and Kaschak (2002) found. When actions were incompatible with the conceptual content of the sentence they were judging, there was an interference effect.

Glenberg and Kaschak do not fully spell out what they have in mind by claiming that language is understood directly in terms of action. One plausible interpretation, however, is that this paradigmatically cognitive task traffics in states with a partially action-oriented representational format—perhaps a motor format specifying the direction of movement. As I said above, this evidence is at least suggestive that cognition does not always map content into a proprietary code, but instead uses the representations given by perceptual and motor systems in a flexible way to solve paradigmatically cognitive tasks. It is plausible to think that the way that this works involves mixed format representational states.

*How* might cognition manage to construct such states? I do not know—that is a question for further research. One salient possibility is this. These representations might be built up over time by processes of integrative or associative learning. Perhaps the cognitive system begins simply by associating contents in one format with contents in another, over time conjoining differently formatted contents in useful ways.

In connection with this option, a recent paper out of Morgan Barense’s lab provides some tantalizing data (Martin et al. 2018). One motivation behind the experiment is, as the experimenters note, the representational-hierarchical model of object coding originally put forward by Murray and Bussey (1999). On this model information processing in the ventral visual stream proceeds hierarchically, with more rostral regions in the stream coding for ‘representations of increasing complexity’ (146). Regarding the more complex representations, Murray and Bussey gave a central role to the perirhinal cortex (PRC), which they claimed performed critical roles in organizing visual features of objects, as well as in organizing semantic features—in virtue of its anatomical location, they argued the PRC ‘allows the linking of representations stored in diverse sensory and motor areas’ (150).

Martin et al. (2018) note that more recent work on the PRC suggests two separate ideas. First, that the PRC plays a critical role for object representations organized in terms of conceptual feature conjunctions. Second, that the PRC plays a critical role for object representations described in terms of visual feature conjunctions. Unfortunately, literatures suggestive of either function are hard to merge due to stimuli that conflate perceptual and conceptual similarity (e.g., horse is conceptually and visually similar to donkey, making it difficult to understand what kind of similarity is responsible for horse-donkey processing features).

To overcome this problem, Martin et al. deployed representational similarity analysis regarding fMRI, with the explicit aim of testing whether and at what place in the brain conceptual features might be integrated with perceptual features. First, they designed sets of stimuli that kept conceptual and perceptual similarity and dissimilarity distinct. Then, they had participants perform property verification tasks on the relevant stimuli. When they analyzed patterns in neural processing profiles across regions of interest, they found the following. Lateral occipital cortex strongly associated with the processing of a visual similarity code. The temporal pole and the parahippocampal cortex was strongly associated with the processing of a conceptual similarity code. And, uniquely, the PRC was strongly associated with the processing of both similarity codes. Further, a more fine-grained spotlight analysis of subsets of voxels in various regions of interest found that a subset of voxels in the PRC ‘simultaneously expressed both visual and conceptual similarity structure’ (11). As Martin et al. infer, this suggests that the PRC supports integration of visual and conceptual features constitutive of an object concept.

Why think this result indicates the existence of a mixed-format psychological state? Because the PRC’s association with both similarity structures occurs no matter the task—visual or conceptual—being performed. It is not as though the PRC is associated with the flexible processing of visual or conceptual similarity structures. It seems, rather, that the PRC traffics in representations that in some way conjoin both structures:

[T]he degree of similarity between multi-voxel activity patterns obtained while participants made conceptual judgments, such as whether a ‘hairdryer’ is man-made or a ‘gun’ is pleasant, was captured by the degree of visual similarity between these object concepts. Likewise, the degree of similarity between multivoxel activity patterns obtained while participants made visual judgments, such as whether a ‘hairdryer’ is angular or a ‘comb’ is elongated, was captured by the degree of conceptual similarity between these object concepts. In both cases, PRC carried information about pre-existing representations of object features that were neither required to perform the immediate task at hand, nor correlated with the features that did in fact have task-relevant diagnostic value. (18)

The right inference to draw is that representations subserved by the PRC—the place where, arguably, object representations become ‘fully specified’ (19)—are mixed-format representations. One might predict that mixed-format representations of actionable objects give rise to mixed-format psychological processes relevant to reasoning about, and action upon and with, such objects.

That evidence concerned mechanisms of association or integration that construct mixed format representation over time. A second option is that formats could be linked together in cognition at a time via a mechanism of stipulation. How exactly this would work, I do not know. But consider how demonstrative thought works. With demonstrative thought, agents are able to link together information presented by perceptual systems with information that resides in conceptual knowledge stores. This capacity gives agents a high degree of flexibility in thinking through how to act upon the items in their immediate surround. Jacob Beck offers an example of what I have in mind here in a recent discussion of perceptually grounded demonstrative thought (PGDT):

[I]f the conceptual attributives in PGDTs were perceptually grounded, they should be proximally constrained in the way that perceptual attributives are. But they’re not. Imagine watching a spotted sandpiper fly across the sky. At first, the sandpiper is close enough that you can see the distinctive pattern of dark spots on its breast. At this point, you can veridically attribute spottedness to the bird in both perception and PGDT. As it flies further away, however, the sandpiper eventually reaches a point where you can still see it, but can no longer make out its spots perceptually. Yet, using a PGDT, you can still veridically think, *That bird is spotted*. (2018, p. 330)

And of course one can think more than just thought about how the bird looks. One can think *That spotted bird is a nasty piece of work,* and so on. Demonstrative thought, then, may function as a stipulative mechanism for conjoining representations in different formats for use in cognition.

*Why* might cognition perform its work in part by constructing and using mixed format representational states? In short, because mixed format states alter the transitional profile of the set of states with which they interact. States with mixed formats can—although, of course, needn’t—alter the transitional profile of a set of psychological states in ways that make the set far more useful than the disjunction of differently formatted states. How a set of mixed format states will do so will depend upon multiple factors—the nature of the mix, the contexts in which such states get used, and so on.

Consider Camp’s (2015) discussion of a kind of state that looks—to me—like a state with a mixed format. Camp calls these states *associative characterizations*. They resemble the states psychologists often call prototype concepts, and Camp explicitly distinguishes them from logically structured contents. As Camp (2015) points out, content-driven transitions between associative characterizations appear to play by different rules than content-driven transitions between logically structured conceptual representations. The reason is that associative characterizations combine a wide range of elements—Camp mentions ‘properties, images, and responses’ (609)—into intuitive wholes. As a result, associative characterizations can combine, but not in the manner of logically structured concepts: ‘just because I have characterizations of two types, or of an individual and a property, it does not follow that I also have a characterization of their combination. For instance, I have a characterization (or stereotype) of bank tellers, and another of feminists, but none of feminist bank tellers’ (609). As a further, very interesting, result, Camp notes that combinations of associative characterizations may produce emergent features—‘I have a characterization of Napoleon, and another of mistresses, and I can form a characterization of Napoleon’s mistress, but it contains many features (hairstyle, dress, personality) that are not part of my characterization of mistresses per se’ (609). Thus, associative characterizations may be problematic in certain contexts, but also far more usable in others.

The claim is not that associative characterizations are structured for the kind of systematic reasoning that classical concepts are thought to be. It is, rather, that such states might enable norm-following or norm-approximation in conditions where efficiency and context-sensitivity are crucial. Recall one of the points made earlier regarding representational formats: different representational formats will enable different manners of transition between states. Some representational formats will be more usable in some contexts than others. In being more usable for action guidance in certain contexts, mixed format states may enable greater norm-sensitivity, even if they are not perfectly structured for logical reasoning.

Consider the use of what Robert Briscoe (2008) calls ‘make-perceive’ (see also Gauker (2011) on ‘imagistic cognition’). Agents often voluntarily or intentionally generate mental imagery in order to augment the current experiential field or to adumbrate nearby possibilities. This imagery may be motoric or involve various perceptual modalities, depending on the needs of the agent. Developing a facility with this capacity is extraordinarily helpful for the smooth control of action. One reason it is so is that the representations at issue are more appropriate for the circumstances than abstract propositions would be. Briscoe (2018) considers the example of a rock-climber. When climbing a wall, a climber needs to execute several sub-actions that balance accuracy of reach, distribution of weight, strength of grip, position of the feet, accessibility and climbability of upcoming patches of rock, and so on. Skilled rock climbers may rely on propositionally structured reasoning at times. But they do not regularly reason in a detached, syllogistic way through all of these factors. Rather, skilled climbers rely on enhanced perceptual recognition of elements on the rock wall, and enhanced abilities to deploy motor and perceptual imagery as they work through the best ways to execute the action of climbing the wall (see Pezzulo et al. 2010). Plausibly, doing so involves assessments of relations of reason between ways of executing the action, goals, one’s own abilities, and available physical resources. Agents may carry out such reasoning dealing only with imagistic resources. But having recourse to propositionally formatted inferences regarding conceptual information embedded within states of make-perceive would certainly be useful.

## Conclusion

I have reviewed results and given arguments in support of four ideas: that [a] cognitive processes of practical reasoning play a key role in the intelligent guidance of action, [b] these processes could not do so without significant enabling work done by both perception and the motor system, [c] the work done by perceptual and motor systems can be characterized as the generation of information (often conceptually structured information) specialized for action guidance, which in turn suggests that [d] the cognitive processes of practical reasoning that play a key role in the guidance of intelligent action are not the abstract, syllogistic ones philosophers often treat as the paradigm of practical reasoning. Rather, these cognitive processes are constrained by, and work well with, the specialized concepts outputted by perception and the feedback outputted by sensorimotor processes. I have not provided a semantics for any mixed format states, nor have I provided a taxonomy of the kinds of mixed format states potentially relevant to intelligent action guidance. Much work, obviously, remains to be done. I have attempted to put results and argumentation together in suggestive ways because it seems to me that a fruitful science of cognition—that is to say, a science of reasoning and intelligent action guidance—may be in part a science of mixed format states, their functional profile, and the types of psychological processes they enable and constrain. If that is right, more explicit attention to the nature of such states and processes is required.

## Footnotes

1 One upshot of this way of thinking about cognition and perception is that use of these terms is consistent with data and usage in the literature on embodied and embedded cognition. ‘Cognition’ may include processes that utilize perceptuo-motor representations, but turned towards paradigmatically cognitive operations. Thanks to a referee for pushing me to clarify this.

2 Incidentally, it is worth noting that this is consistent with the view that perception is encapsulated from cognition, as well as the view that a rapid perceptuo-motor system is functionally specialized for perceptuo-motor transformations (Goodale 1988; Pylyshyn 2003). What it suggests, however, is that the outputs of perception, and perhaps some of the feedback outputs of perceptuo-motor processes, arrive *to* cognition primed for efficient use *by* cognition. If the formats of the relevant states were not usable by cognition, cognitively generated action intentions would not engage spatial attention in the ways that they do, nor would cognitively generated action templates guide visual search in the way that they can.

3 In unpublished work, Ellen Fridland has been developing a complimentary perspective on the importance of reasoning in action guidance (Fridland n.d.).

4 Work on the content of pictures is, however, relevant (see, e.g., Grzankowski 2015), as is work on representational formats in general (Camp 2007; Rescorla 2009; Beck 2015; Green and Quilty-Dunn 2017).
